# Causes and Characteristics of Children Unintentional Injuries in Emergency Department and Its Implications for Prevention

**DOI:** 10.3389/fpubh.2021.669125

**Published:** 2021-08-05

**Authors:** Hairong Gong, Guoping Lu, Jian Ma, Jicui Zheng, Fei Hu, Jing Liu, Jun Song, Shenjie Hu, Libo Sun, Yang Chen, Li Xie, Xiaobo Zhang, Leilei Duan, Hong Xu

**Affiliations:** ^1^Emergency Department, Children's Hospital of Fudan University, Shanghai, China; ^2^Department of Neurosurgery, Children's Hospital of Fudan University, Shanghai, China; ^3^Department of Orthopedics, Children's Hospital of Fudan University, Shanghai, China; ^4^Clinical Research Institute, Shanghai Jiao Tong University School of Medicine, Shanghai, China; ^5^Department of Respiratory, Children's Hospital of Fudan University, Shanghai, China; ^6^Division of Injury Prevention and Mental Health, National Center for Chronic and Non-communicable Disease Control and Prevention, Chinese Center for Disease Control and Prevention, Beijing, China; ^7^Department of Nephrology, Children's Hospital of Fudan University, Shanghai, China

**Keywords:** unintentional injury, children, characteristics, emergency department, prevention

## Abstract

**Background:** Child unintentional injuries have become a hot topic worldwide, and substantial regional disparities existed in causes and characteristics. To date, limited data are available to investigate the causes and characteristics of child unintentional injuries from hospitals for children in China.

**Methods:** A cross-sectional study was conducted between January 2017 and December 2018 in Shanghai, China. Patients aged <18 years with an unintentional injury presented to the emergency department were enrolled. Demographic information, Pediatric Risk for Mortality III score, and outcome variables were retrieved from electronic health records (EHRs). Frequencies and proportions of categorical variables and means and SDs of continuous variables are presented. Chi-square test and Student's *t*-test were used for the comparison between groups, as appropriate. Logistic regression analysis was used to estimate potential risk factors for admission to the hospital.

**Results:** A total of 29,597 cases with unintentional injuries were identified between January 2017 and December 2018, with boys vs. girls ratio of 1.75. Preschool children account for approximately two-thirds of unintentional injuries in the emergency department. A distinctive pattern of mechanisms of unintentional injuries between gender was documented, and sports injury was significantly higher in boys than in girls (10.2 vs. 7.8%). Compared with Canadian Emergency Department Triage and Acuity Scale (CTAS) Grade 3 patients, Grade 2 [odds ratio (OR) = 2.99, 95% CI = 1.93–4.63, *P* < 0.001] and Grade 1 (OR = 74.85, 95% CI = 12.93–433.14, *P* < 0.001) patients had higher risk of inhospital admission. For causes of injuries, compared with falling, foreign body and poison had a lower risk of inhospital admission, while transport injury (OR = 1.31, 95% CI = 1.07–1.59, *P* = 0.008) and high fall injury (OR = 2.58. 95% CI =1.48–4.49, *P* < 0.001) had a significantly higher risk of admission.

**Conclusions:** There was a significant relationship between age-groups and unintentional injuries between gender, with decreased injuries among girls growing up older. Preventive measures should be taken to reduce transport injury and high fall injury, which had a significantly higher risk of admission.

## Background

Unintentional injuries have become a hot topic worldwide in public health due to the high disability and mortality. Compared with adults, children and adolescents are at higher risk of suffering from unintentional injuries (1, 2). Unintentional injury is one of the leading causes of death for children of all ages; in 2013, it nearly accounted for 15.4% of the 2.6 million deaths of children aged 1–14 years (1, 3). In 2017, more than 6 million children and young adults were injured seriously enough who need to visit a hospital emergency department (4). Unintentional injuries are also a primary cause of disability, which can have a lasting impact on all aspects of the lives of the children, namely, relationships, learning, and games. The severe injury also leads to emotional trauma and financial burden for families and society (5). Caring for the unintentionally injured child requires specialized knowledge, precise management, and scrupulous attention to detail.

There are substantial regional disparities in causes and characteristics of unintentional pediatric injuries. Previous epidemiologic studies conducted in the United States have reported that unintentional falls are the leading cause of non-fatal injury in children presenting to hospital emergency departments, followed by being struck by or against an object (6). Another study in Nepal reported that falls are the most common cause of unintentional injuries among children (6). Due to the rapid development of the economics of society and the widespread use of comprehensive vaccination programs, the healthcare of children in China has changed significantly over the past 50 years. Although the hospital-based passive surveillance of the national injury surveillance system of medical and health institutions has been well established in China, the monitoring hospitals were not stratified according to hospital for children (7). Considering the majority of younger patients tend to visit the hospitals for children (8), the unstratified national injury surveillance for the hospital for children might cause a potential bias on the report of unintentional pediatric injuries.

A comprehensive assessment of causes and characteristics of child unintentional injury is an essential foundation for selecting and formulating injury prevention strategies and improving emergency and acute care services for injured children. To date, limited data are available to investigate the causes and characteristics of child unintentional injuries from hospitals for children in China. Therefore, we conducted a cross-sectional study to investigate the epidemiologic characteristics and causes of the unintentionally injured child presenting to the emergency department, which is expected to serve as the basis for a piece of in-depth evidence for the child injury prevention control measures.

## Methods

### Study Design and Participants

A cross-sectional, retrospective study was conducted on patients who visited the Emergency Department of Children's Hospital of Fudan University from January 2017 to December 2018. We included children and adolescents aged under 18 years, with a diagnosis of unintentional injury. An unintentional injury is defined as “an unexpected, unintentional, and violent event, affecting a child, with or without detectable lesions, and subsequently leading to medical attention; the injury may have been caused by a fall, poisoning, drowning, burns, or traffic-related accidents.” We excluded all patients with intentional injuries, such as abuse. The study protocol was approved by the Institutional Review Board and Ethics Committee of Children's Hospital of Fudan University and waived the need for the informed consent of parents due to the retrospective nature of the study design. This study was conducted in accordance with the Declaration of Helsinki and reported in accordance with the Strengthening the Reporting of Observational Studies in Epidemiology (STROBE) guidelines.

### Data Collection and Measurement

Unintentional injuries were identified by using *The International Classification of Diseases* (ICD-10) codes: S00-S99, T00-T98, V01-V99, W00-W90, X00-X99, and Y00-Y34, which included motor vehicle crash injuries, falls, drowning, poisoning, suffocation, and animal bites. Data were collected based on the inclusion and exclusion criteria, demographics, clinical characteristics, and outcome measures. The medical records and rescue register records of the emergency children were reviewed in detail, and the admission records were reviewed through the electronic health records (EHRs) to determine their outcome.

### Statistical Analysis

Descriptive statistics were calculated first, including means, SDs, and percentages. Frequencies and proportions of categorical variables and means and SDs of continuous variables are presented. The chi-square test was used to explore the significance of differences between groups, and Student's *t*-test was used for the comparison of continuous variables. To explore the risk factors of clinical outcome, unconditional logistic regression models were used to assess the associations between potential factors and outcomes after the emergency. All statistical procedures were performed using R software (version 3.4.4). A two-sided *P* < 0.05 was considered statistically significant.

## Results

### Overall Characteristics of Patients With Unintentional Injuries

As shown in Table 1, a total of 29,597 cases with unintentional injuries were identified between January 2017 and December 2018, with boys vs. girls ratio of 1.75. The mean age of patients with unintentional injuries was 5.15 ± 3.79 (mean ± SD). There was a significant relationship between age-groups and injuries between gender, with decreased injuries among girls when growing up older. Preschool children under the age of 6 years old account for approximately 64.6% of unintentional injuries in the emergency department (Table 2).

**Table 1:** Characteristics of child unintentional injury patient in the emergency department.

	**Male**	**Female**	**Overall**	***P-*Value**
	**(*n* = 18,816)**	**(*n* = 10,781)**	**(*n* = 29,597)**	
**Age** **(Mean ± SD)**	5.52 ± 3.93	4.51 ± 3.43	5.15 ± 3.79	<0.001
**Age Group**				
Infant	1,760 (9.4%)	1,401 (13.0%)	3,161 (10.7%)	<0.001
Child	4,793 (25.5%)	3,205 (29.7%)	7,998 (27.0%)	
Preschool	4,894 (26.0%)	3,079 (28.6%)	7,973 (26.9%)	
School age	5,785 (30.7%)	2,738 (25.4%)	8,523 (28.8%)	
Puberty	1,584 (8.4%)	358 (3.3%)	1,942 (6.6%)	
**Source**				0.144
Ambulance	466 (2.5%)	238 (2.2%)	704 (2.4%)	
By self	18,350 (97.5%)	10,543 (97.8%)	28,893 (97.6%)	
**Injury hours (Mean ± SD)**	11.1 (16.9)	11.2 (18.4)	11.1 (17.4)	0.486
**GCS Total Point**				0.938
<8	18 (0.1%)	10 (0.1%)	28 (0.1%)	
≥8	18,798 (99.9%)	10,771 (99.9%)	29,569 (99.9%)	
**Consciousness**				0.908
Conscious	18,795 (99.9%)	10,770 (99.9%)	29,565 (99.9%)	
Slow Conscious	11 (0.1%)	5 (0.0%)	16 (0.1%)	
Coma	10 (0.1%)	6 (0.1%)	16 (0.1%)	
**Prehospital Procedure**				0.458
Yes	127 (0.7%)	65 (0.6%)	192 (0.6%)	
No	18,689 (99.3%)	10,716 (99.4%)	29,405 (99.4%)	
**Combined Other Organ Injuries**				0.533
Yes	15 (0.1%)	11 (0.1%)	26 (0.1%)	
No	18,801 (99.9%)	10,770 (99.9%)	29,571 (99.9%)	
**Ventilation**				0.950
Yes	5 (0.0%)	3 (0.0%)	8 (0.0%)	
No	18,811 (100.0%)	10,778 (100.0%)	29,589 (100.0%)	
**After Emergency**				<0.001
Go home	17,076 (90.8%)	9,930 (92.1%)	27,006 (91.2%)	
Admission	1,692 (9.0%)	819 (7.6%)	2,511 (8.5%)	
ICU Admission	48 (0.3%)	32 (0.3%)	80 (0.3%)	

*SD, Standard deviation; GCS, Glasgow coma scale; ICU, Intensive care unit*.

**Table 2:** Characteristics of child unintentional injury patient in the emergency department by age-group.

	**Infant (0–1 years)**	**Child (1–3 years)**	**Preschool (3–6 years)**	**School-age (6–12 years)**	**Puberty (12–18 years)**	***P-*Value**
	**(*n* = 3,161)**	**(*n* = 7,998)**	**(*n* = 7,973)**	**(*n* = 8,523)**	**(*n* = 1,942)**	
**Source**						<0.001
Ambulance	70 (2.2%)	164 (2.1%)	184 (2.3%)	193 (2.3%)	93 (4.8%)	
By self	3,091 (97.8%)	7,834 (97.9%)	7,789 (97.7%)	8,330 (97.7%)	1,849 (95.2%)	
**Injury hours (Mean ± SD)**	8.12 (15.3)	9.65 (17.2)	10.6 (16.0)	13.2 (18.4)	15.2 (21.0)	<0.001
**GCS Total Point**						0.338
<8	5 (0.2%)	8 (0.1%)	5 (0.1%)	10 (0.1%)	0 (0%)	
≥8	3,156 (99.8%)	7,990 (99.9%)	7,968 (99.9%)	8,513 (99.9%)	1,942 (100%)	
**Consciousness**						0.180
Conscious	3,156 (99.8%)	7,992 (99.9%)	7,963 (99.9%)	8,512 (99.9%)	1,942 (100%)	
Slow Conscious	2 (0.1%)	3 (0.0%)	8 (0.1%)	3 (0.0%)	0 (0%)	
Coma	3 (0.1%)	3 (0.0%)	2 (0.0%)	8 (0.1%)	0 (0%)	
**Prehospital procedure**						<0.001
Yes	17 (0.5%)	38 (0.5%)	57 (0.7%)	52 (0.6%)	28 (1.4%)	
No	3,144 (99.5%)	7,960 (99.5%)	7,916 (99.3%)	8,471 (99.4%)	1,914 (98.6%)	
**Multiple Organ Injuries**						0.773
Yes	2 (0.1%)	8 (0.1%)	9 (0.1%)	5 (0.1%)	2 (0.1%)	
No	3,159 (99.9%)	7,990 (99.9%)	7,964 (99.9%)	8,518 (99.9%)	1,940 (99.9%)	
**Ventilation**						0.837
Yes	1 (0.0%)	3 (0.0%)	1 (0.0%)	2 (0.0%)	1 (0.1%)	
No	3,160 (100.0%)	7,995 (100.0%)	7,972 (100.0%)	8,521 (100.0%)	1,941 (99.9%)	
**After emergency**						<0.001
Go home	3,094 (97.9%)	7,662 (95.8%)	7,238 (90.8%)	7,421 (87.1%)	1,591 (81.9%)	
Admission	57 (1.8%)	312 (3.9%)	712 (8.9%)	1,082 (12.7%)	348 (17.9%)	
ICU admission	10 (0.3%)	24 (0.3%)	23 (0.3%)	20 (0.2%)	3 (0.2%)	

*SD, Standard deviation; GCS, glasgow coma scale; and ICU, intensive care unit.

### Mechanisms of Unintentional Injuries by Gender

Unintentionally fall was the most common reason for unintentional injuries among both the gender, accounting for 72.2% cases of unintentional injury, with 71.8% and 72.9% for boys and girls, respectively (Table 3, Figure 1). Following fall, the frequency of observed mechanism of injury was sports (9.3%), foreign body (6.4%), struck (4.9%), transport (4.1%), poison (1.2%), cut or pierce (0.9%), and high fall (0.4%). A distinctive pattern of mechanisms of unintentional injuries between gender was documented, and sports were significantly higher in boys than in girls (10.2 vs. 7.8%).

**Table 3:** Mechanism of child unintentional injury in the emergency department.

	**Male**	**Female**	**Overall**	***P-*Value**
	**(*n* = 18,816)**	**(*n* = 10,781)**	**(*n* = 29,597)**	
**Mechanism of injury**				<0.001
Fall	13,507 (71.8%)	7,856 (72.9%)	21,363 (72.2%)	
Sports	1,923 (10.2%)	842 (7.8%)	2,765 (9.3%)	
Foreign body	1,167 (6.2%)	728 (6.8%)	1,895 (6.4%)	
Struck	927 (4.9%)	518 (4.8%)	1,445 (4.9%)	
Transport^*^	758 (4.0%)	468 (4.3%)	1,226 (4.1%)	
Cut or pierce	157 (0.8%)	105 (1.0%)	262 (0.9%)	
Poison	204 (1.1%)	159 (1.5%)	363 (1.2%)	
High Fall	80 (0.4%)	41 (0.4%)	121 (0.4%)	
**Position of injury**				<0.001
Head	9,057 (48.1%)	5,285 (49.0%)	14,342 (48.5%)	
Body	327 (1.7%)	255 (2.4%)	582 (2.0%)	
Limbs	7,917 (42.1%)	4,246 (39.4%)	12,163 (41.1%)	
Not Available	1,368 (7.3%)	886 (8.2%)	2,254 (7.6%)	
**Protection^#^**				<0.001
Yes	18 (2.37%)	4 (0.85%)	22 (1.79%)	
No	740 (97.63%)	464 (99.15%)	1,204 (98.21%)	

* *Transport including pedestrian, pedal cyclist, and motorcyclist*.

# *For transport injury only*.

**Figure 1:**
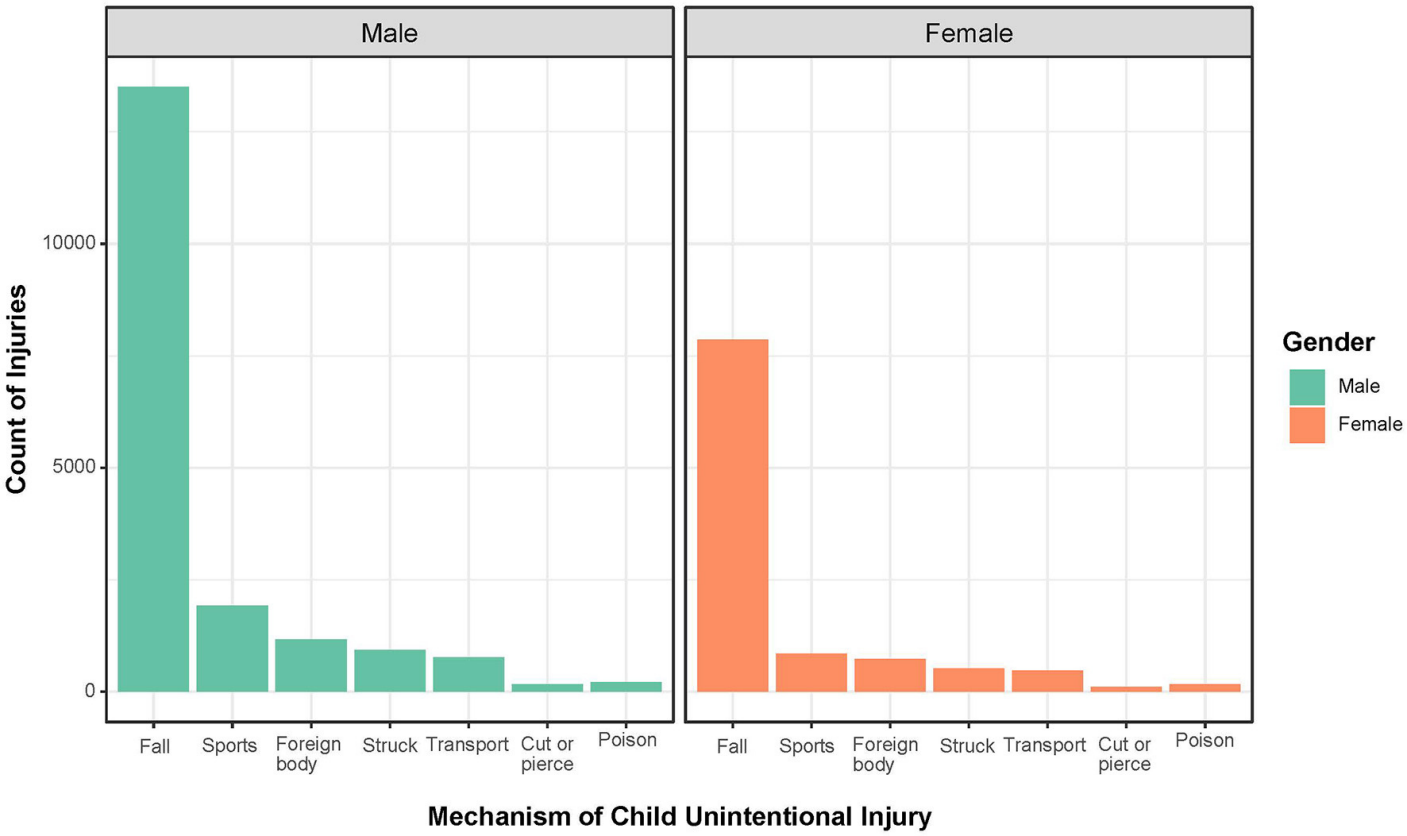
Mechanism of child unintentional injuries by gender in the emergency department. Gender was colored by green (boys) and orange (girls). X row represents the mechanism of child unintentional injuries. Y row represents the count of injuries.

### Mechanisms of Unintentional Injuries by Age-Groups

There was a significantly different pattern of the mechanism of injury among age-groups (Table 4, Figure 2). As they grow up older, there was a decreased prevalence of falls among age-groups, with 85.8% among 0–1 year old vs. 49.7% among 12–18 years of puberty. Fall (85.5%), foreign body (7.4%), and struck (1.5%) were the top three reasons for unintentional injury among 0–1-year age-group. Among the age-group of 1–3 years, the common reasons for unintentional injury were fall (77.7%), foreign body (10.2%), and transport (3.6%). Similarly, in the preschool group (aged 3–6 years), fall (76.8%), foreign body (6.4%), and transport (6.0%) were the top three reasons for unintentional injury. Among school-age (6–12 years) and puberty (12–18) groups, the top three reasons for unintentional injury were fall, sports, and transport.

**Table 4:** Mechanism of child unintentional injury in the emergency department by age-group.

	**Infant (0–1 years)**	**Child (1–3 years)**	**Preschool (3–6 years)**	**School age (6–12 years)**	**Puberty (12–18 years)**	***P* Value**
	**(*n* = 3,161)**	**(*n* = 7,998)**	**(*n* = 7,973)**	**(*n* = 8,523)**	**(*n* = 1,942)**	
**Mechanism of injury**						<0.001
Fall	2,711 (85.8%)	6,213 (77.7%)	6,124 (76.8%)	5,349 (62.8%)	966 (49.7%)	
Sports	8 (0.3%)	79 (1.0%)	257 (3.2%)	1,711 (20.1%)	710 (36.6%)	
Foreign body	235 (7.4%)	817 (10.2%)	511 (6.4%)	306 (3.6%)	26 (1.3%)	
Struck	46 (1.5%)	244 (3.1%)	353 (4.4%)	638 (7.5%)	164 (8.4%)	
Transport^*^	39 (1.2%)	284 (3.6%)	480 (6.0%)	367 (4.3%)	56 (2.9%)	
Cut or pierce	35 (1.1%)	61 (0.8%)	81 (1.0%)	77 (0.9%)	8 (0.4%)	
Poison	34 (1.1%)	199 (2.5%)	98 (1.2%)	28 (0.3%)	4 (0.2%)	
High Fall	18 (0.6%)	41 (0.5%)	41 (0.5%)	18 (0.2%)	3 (0.2%)	
**Position of injury**						<0.001
Head	2,595 (82.1%)	4,909 (61.4%)	3,736 (46.9%)	2,831 (33.2%)	271 (14.0%)	
Body	42 (1.3%)	173 (2.2%)	188 (2.4%)	138 (1.6%)	41 (2.1%)	
Limbs	191 (6.0%)	1,803 (22.5%)	3,397 (42.6%)	5,177 (60.7%)	1,595 (82.1%)	
Not Available	270 (8.5%)	1,013 (12.7%)	607 (7.6%)	334 (3.9%)	30 (1.5%)	
**Protection^#^**						0.321
Yes	1 (2.56%)	3 (1.06%)	7 (1.46%)	10 (2.72%)	1 (1.79%)	
No	38 (97.44%)	281 (98.94%)	473 (98.54%)	357 (97.28%)	55 (98.21%)	

* *Transport including pedestrian, pedal cyclist, and motorcyclist*.

# *For transport injury only*.

**Figure 2:**
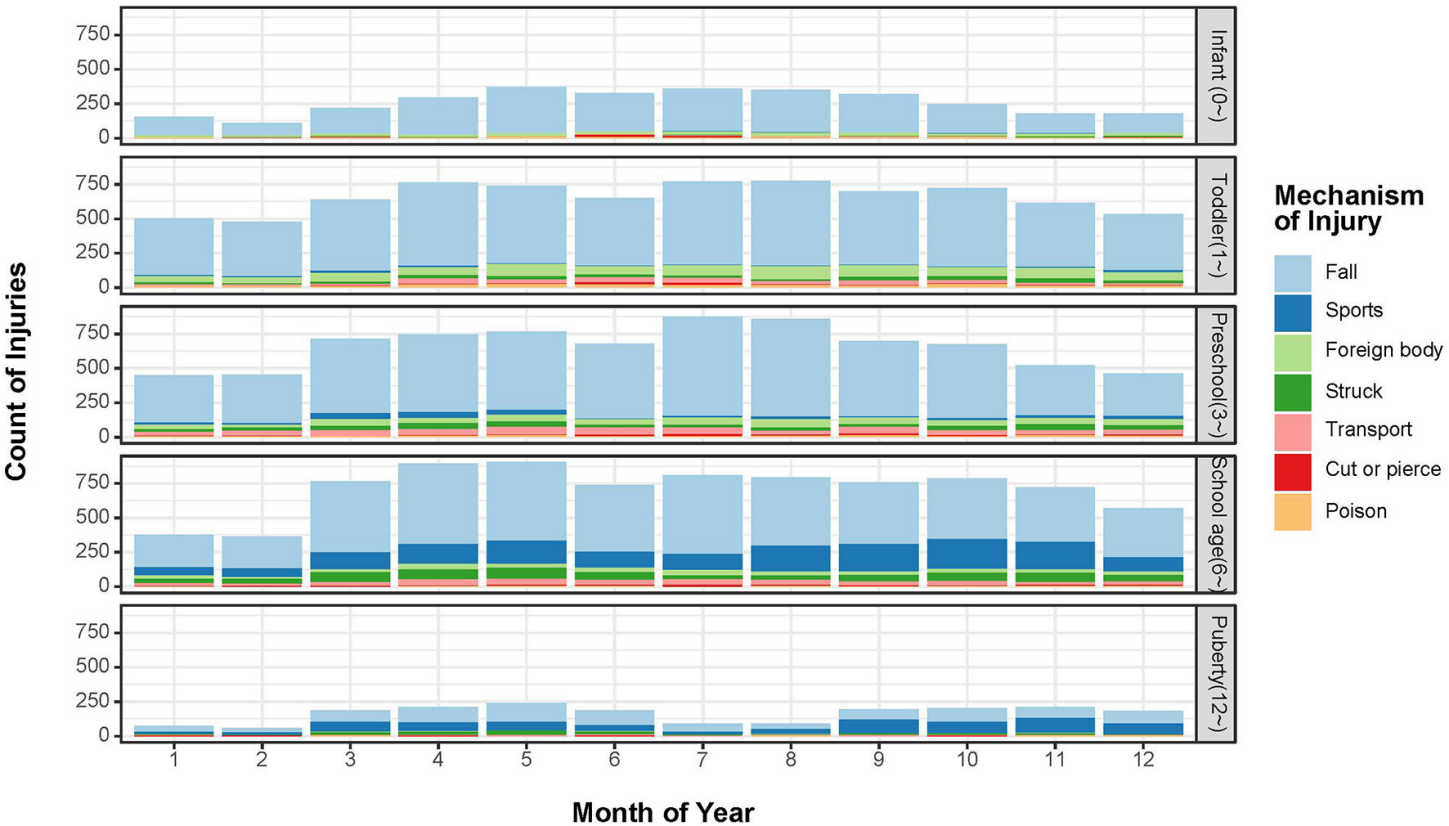
Time trends of child unintentional injury mechanisms by age-group. X row represents the month of the year. Y row represents the count of injuries. Child unintentional injury mechanisms were stacked by different colors.

### Association Between Characteristics of Unintentional Injuries and Clinical Outcomes

We analyze the association between characteristics of unintentional injuries and inhospital admission by multivariable logistic regression. We found that there was no statistical difference between male and female patients. Patients transferred by ambulance had elevated the risk of inhospital admission than the patient who came by themselves [odds ratio (OR) = 2.30, 95% CI = 1.51–3.51, *P* < 0.001]. Compared with Grade 3 of CTAS patients in the emergency department, Grade 2 (OR = 2.99, 95% CI = 1.93–4.63, *P* < 0.001) and Grade 1 of CTAS (OR = 74.85, 95% CI = 12.93–433.14, *P* < 0.001) patients had statistically higher risk of inhospital admission. For different mechanisms of injuries, compared with fall, foreign body (OR = 0.37, 95% CI = 0.27–0.51, *P* < 0.001) and poison (OR = 0.10, 95% CI = 0.04–0.29, *P* < 0.001) had lower risk of inhospital admission, while transport (OR = 1.31, 95% CI = 1.07–1.59, *P* = 0.008) and high fall (OR = 2.58. 95% CI = 1.48–4.49, *P* < 0.001) had a significantly higher risk of admission. The results of the multivariate analysis are shown in Table 5.

**Table 5:** Risk factors of admission in hospital among child unintentional injury patient in the emergency department.

**Factors**		OR^*^	**95% CI**	***P-*Value**
**Age**		1.14	(1.13,1.15)	<0.001
**Gender**	Girl	1.00	(Reference)	-
	Boy	1.01	(0.92,1.12)	0.785
**Source**	By self	1.00	(Reference)	-
	Ambulance	2.30	(1.51,3.51)	<0.001
**CTAS^#^ grade**	Grade 3	1.00	(Reference)	-
	Grade 2	2.99	(1.93,4.63)	<0.001
	Grade 1	74.85	(12.93,433.14)	<0.001
**Injury time (h)**		1.00	(0.99,1.00)	0.017
**Prehospital procedure**	No	1.00	(Reference)	-
	Yes	2.07	(1.40,3.08)	<0.001
**Mechanism of injury**	Fall	1.00	(Reference)	-
	Sports	1.05	(0.90,1.21)	0.546
	Foreign body	0.37	(0.27,0.51)	<0.001
	Struck	1.19	(0.99,1.43)	0.068
	Transport	1.31	(1.07,1.59)	0.008
	Cut or pierce	1.05	(0.57,1.91)	0.882
	Poison	0.10	(0.04,0.29)	<0.001
	High Fall	2.58	(1.48,4.49)	0.001
**Multiple organ injuries**	No	1.00	(Reference)	-
	Yes	44.04	(5.54,350.12)	<0.001

* *OR, Odds ratios; CI, confidence Interval*.

# *CTAS, Canadian Emergency Department Triage and Acuity Scale*.

## Discussion

In this study, we observed the variation in causes and characteristics of child unintentional injury by age-group and gender in the emergency department. Preschool children under the age of 6 years old account for approximately 64.6% of unintentional injuries in the emergency department. The CTAS Grade 2 and Grade 1 patients had a statistically higher risk of inhospital admission.

Among all age-group, our study found that the proportion of unintentional injuries was significantly higher in boys than girls, which is in agreement with the previous studies (9). The elevated risk might associate with a higher level of activates and a different pattern of behaviors among boys and girls. Consistent with the previous studies, unintentional falls are the leading cause of non-fatal injuries in emergency department visits among children under the age of 14 years old (10–12). A previous study that has examined falls in children provided some information on young children who experience fall injuries and the circumstances of the fall. Compared to older children, children under the age of 5 years old are more likely to sustain head injuries, be hospitalized, or die from falls (13). Child protection strategies have been widely recognized and publicly supported. Child unintentional injuries are one of the most prominent global health threats, and parents may play a vital role in preventing these injuries. There has been speculation that the one-child policy has led to greater parental attention to children, thus resulting in considerable improvements in the younger age-groups, as many injuries may be due to a lack of supervision or dangerous circumstances (14, 15). In addition, the behaviors of children should be taken into consideration when developing prevention measures.

Most unintentional injuries are mild and do not require extra treatment such as inhospital admission. However, a small proportion of patients might present as having mild injuries but have clinically significant injuries. Although CT provides a definitive and rapid diagnosis to confirm or exclude intracranial injuries, there is concern about radiation-induced cancer, particularly in young patients. A national-wide injury surveillance study found that although the incidence of injuries increased between 1990 and 2017 in China, the injury burden in terms of mortality and disability-adjusted life year greatly improved (16).

To better perform prevention measures of unintentional injuries in children, it is essential to take more consideration of child development and parental supervision when designing prevention efforts. Prevention of unintentional injury for children should be included in all survival, maternal, and child health programs and integrated into all primary healthcare systems, both in high-income and low-income countries or intermediate-income countries. In China, with the improvement of the living conditions and the development of healthcare, the deaths of children caused by infectious diseases have been effectively controlled and unintentional injuries are gradually becoming the main cause of death among children under the age of 5 years old (17, 18). In addition to injury prevention, programs should improve emergency and acute care services for the injured children and strengthen rehabilitation programs. Also, parents teaching safety rules could reduce risks of unintentional child injuries and modestly improve child safety behaviors (19).

### Strengths and Weaknesses of the Study

Our study had some strengths, such as collecting data from the emergency department is a reliable and efficient way to obtain information on the unintentional injury of children and has a relatively large sample size. This study had several limitations. First, this cross-sectional study in nature had no long-time follow-up of unintentional injuries patients. Outcomes were determined by a review of EHRs in detail. Second, we failed to collect data on risk factors related to unintentional injuries, such as physical and mental health conditions of children before the accident, family characteristics, family income, and educational level of parents. As a result, we cannot quantify the other risk factors for unintentional injuries. Future studies should measure these factors relating to unintentional injuries among children more explicitly. Third, our study was based on the data of a single hospital, which might restrict the generalizability of findings from this study.

## Conclusions

Our study found that significant variation exists in causes and characteristics of child unintentional injury by age-group and gender in the emergency department. There was a significant relationship between age-groups and unintentional injuries between gender, with decreased injuries among girls growing up older. Preventive measures should be taken to reduce transport injury and high fall injury, which had a significantly higher risk of admission.

## Data Availability Statement

The raw data supporting the conclusions of this article are available from the corresponding author by request.

## Ethics Statement

The studies involving human participants were reviewed and approved by Institutional Review Board and Ethics Committee of Children's Hospital of Fudan University. Written informed consent from the participants' legal guardian/next of kin was waived due to the retrospective nature of the study design, which in accordance with the national legislation and the institutional requirements.

## Author Contributions

HG and HX conceptualized and designed the study, coordinated and supervised data collection, contributed to data interpretation, drafted the initial manuscript, and reviewed and revised the manuscript. GL, JM, JZ, FH, JL, JS, SH, LS, YC, and LX designed the data collection instruments and collected data. LX carried out the statistical and formal analysis and interpretation. XZ and LD critically reviewed the manuscript for important intellectual content. All authors revised the manuscript and approved the final manuscript as submitted and agreed to be accountable for all aspects of the work.

## Conflict of Interest

The authors declare that the research was conducted in the absence of any commercial or financial relationships that could be construed as a potential conflict of interest.

## Publisher's Note

All claims expressed in this article are solely those of the authors and do not necessarily represent those of their affiliated organizations, or those of the publisher, the editors and the reviewers. Any product that may be evaluated in this article, or claim that may be made by its manufacturer, is not guaranteed or endorsed by the publisher.

## Abbreviations

EHR: Electronic health records
CTAS: Canadian Emergency Department Triage and Acuity Scale
ICD-10: The International Classification of Diseases 10
OR: Odds ratio
CI: Confidence interval
SD: Standard deviations.
